# The association between preoperative chronic pain and postoperative delirium in elderly patients undergoing elective orthopedic surgery

**DOI:** 10.3389/fmed.2026.1795717

**Published:** 2026-03-23

**Authors:** Qianyu Yang, Jiawei Han, Lanyan Xue, Yanan Zhao, Lu Chen, Zixuan Wang, Jiayu Zhu, Xuesen Su, Shouyuan Tian

**Affiliations:** 1 The College of Anesthesia, Shanxi Medical University, Taiyuan, Shanxi, China; 2 Cancer Hospital Affiliated to Shanxi Medical University, Shanxi Medical University, Taiyuan, Shanxi, China; 3 The Department of Anesthesiology, The Fifth Clinical Medical College of Shanxi Medical University, Taiyuan, Shanxi, China; 4 Shanxi Key Laboratory of Geriatric Precision Anesthesia and Complication Prevention, The Fifth Clinical Medical College of Shanxi Medical University, Taiyuan, Shanxi, China

**Keywords:** chronic pain, geriatric patients, pain intensity, postoperative delirium, risk factors

## Abstract

**Background:**

Postoperative delirium (POD) is an acute and common postoperative complication that can increase morbidity and mortality. The evidence regarding the association between preoperative chronic pain and POD is inconsistent. This study aims to explore the relationship between preoperative chronic pain and POD.

**Methods:**

This prospective cohort study consecutively enrolled 200 elderly patients undergoing elective orthopedic surgery. POD occurring within 7 days was assessed using the 3-min Diagnostic Interview for Confusion Assessment Method (3D-CAM). Chronic pain was defined as pain lasting ≥ 3 months, with intensity assessed by VAS preoperatively. Demographic and perioperative baselines were compared between delirium and non-delirium groups. Univariate and multivariate logistic regression were used to identify POD risk factors. Subgroup analyses focus on chronic pain patients were performed to explore the risk factors of developing POD among them.

**Results:**

Among the 200 enrolled patients (median age, 69 years; ASA II/III, 106/94), POD occurred in 33% (66/200) and preoperative chronic pain occurred in 58.5% (117/200). Univariate and multivariable logistic regression analyses showed significant association between preoperative chronic pain and POD among elder population (unadjusted OR = 2.039, 95% CI: 1.090–3.811; adjusted OR = 2.488, 95% CI: 1.282–4.837, *P* = 0.007). A subgroup analysis of chronic pain patients revealed that current pain intensity (VAS scores) was associated with the increased risk of POD (OR = 1.858, 95% CI: 1.291–2.675, *P* < 0.001).

**Conclusion:**

Preoperative chronic pain is associated with the increased risk of POD in the elderly patients undergoing elective orthopedic surgery. Furthermore, a significant association between current pain intensity assessed by VAS and POD was found among older adults with chronic pain.

## Introduction

Postoperative delirium (POD) is a common complication among elderly patients, characterized by acute fluctuations in mental status, consciousness, attention, cognition, and perception, typically occurring within the first 1–3 days after surgery (1, 2). According to previous research, its incidence ranges from 11 to 51% in non-cardiac surgeries, with a higher frequency observed in orthopedic patients, reaching approximately 50% following hip or knee arthroplasty (3–5). POD contributes to a range of adverse clinical outcomes including prolonged hospital stays (6), elevated readmission rates, and increased mortality (7). These unfavorable consequences not only impose a substantial burden on medical resources and healthcare finances but also exert a broader impact on society as a whole, with no effective intervention currently available. Therefore, the clinical significance of managing POD during the preoperative period is underscored.

Chronic pain—defined as pain persisting beyond 3 months (8)—is highly prevalent in the general population, often leading to impaired physical function, compromised quality of life, and even disability (9). As a major public health challenge, its estimated prevalence reaches 74% among the geriatric orthopedic population (10). Several cohort studies have demonstrated that chronic pain is associated with memory decline, accelerated cognitive deterioration (11), and an increased risk of cognitive impairment (12, 13).

Previous findings regarding the relationship between preoperative chronic pain and POD have been inconsistent. While several studies reported that higher levels of preoperative pain increase the risk to develop POD (14–17), others have found this association only in bivariate analyses (18–20), and some have not found an association at all (21–23). These discrepancies may be due to differences in study populations. Notably, this relationship in specific populations, such as geriatric orthopedic patients, has not been further investigated.

Therefore, this study aimed to investigate the relationship between preoperative chronic pain and POD in elderly patients undergoing elective orthopedic surgery. We hypothesized that chronic pain may represent a modifiable risk factor and a potential target for intervention.

## Materials and methods

### Study design and population

This prospective cohort study consecutively enrolled all patients at our hospital from April to July of 2025. The inclusion criteria required that patients be scheduled for elective orthopedic procedures, specifically total knee arthroplasty, total hip arthroplasty, or hemiarthroplasty, at our hospital between April and July 2025. Eligible participants had to be at least 65 years of age and classified as American Society of Anesthesiologists (ASA) Physical Status I-III. Additionally, they were required to demonstrate unimpaired communication abilities and the capacity to complete preoperative assessment scales. Exclusion criteria consisted of a pre-existing diagnosis of malignancy or a life expectancy of less than 12 months. Patients with preoperative cognitive disorders, such as Alzheimer’s disease or dementia as defined by DSM-5 criteria, were also excluded. Further exclusion grounds included an inability to complete preoperative evaluations or provide informed consent. Patients transferred directly to the intensive care unit (ICU) following surgery were to be withdrawn from the study.

### Ethical approval and registration

The study was approved by the Institutional Review Board of our institution (Approval Number KYLL-2025-100), and was conducted in accordance with the principles of the Declaration of Helsinki. The trial was registered with the Chinese Clinical Trial Registry (ChiCTR2500105534), and this is a retroactive registration. Written informed consent was obtained from all participants.

### Data collection

Baseline demographic and clinical characteristics collected during preoperative assessment included gender, age, body mass index (BMI), history of alcohol consumption, smoking status, complications, ASA PS, and educational attainment. Especially, all participants showed no evidence of delirium during preoperative evaluations. Postoperatively, surgical parameters extracted from medical records included type of procedure, operative duration, anesthesia time, anesthesia modality (categorized as general or spinal), use of patient-controlled analgesia (PCA), length of stay in the post-anesthesia care unit (PACU), and total hospitalization days.

### Preoperative pain assessment

Chronic pain is conventionally defined as pain persisting for more than 3 months (8). During preoperative assessment, patients were systematically evaluated for the presence of chronic pain. We asked patients whether they experienced chronic pain lasting more than 3 months. All patients reporting preoperative chronic pain lasting ≥ 3 months underwent assessment using the Visual Analog Scale (VAS) to quantify their current pain intensity. The VAS consists of a horizontal line, typically 10 cm in length, with verbal descriptors anchored at both ends. The left end (0 cm) is labeled “0,” representing “no pain,” while the right end (10 cm) is labeled “10,” signifying “the worst imaginable pain” or “most severe pain imaginable.” During administration, the side of the scale with numerical gradations was positioned away from the patient. Participants were instructed to mark a point on the line that corresponded to their perceived pain level. The VAS score (ranging from 0 to 10) was then calculated by measuring the distance (in centimeters) from the “no pain” anchor (0 cm) to the patient’s mark, and recorded to one decimal place. Although the intensity of preoperative chronic pain may be fluctuant, we required patients to use VAS to describe the average intensity of their current pain over the past week to account for daily fluctuations. In addition, patients without preoperative chronic pain or with a pain duration of less than 3 months were identified as non-preoperative chronic pain cases, excluded from the pain group, and not subjected to pain assessment.

### POD assessment

POD was assessed using the Chinese version of the 3-Min Diagnostic Interview for CAM-defined Delirium (3D-CAM), which is primarily utilized for delirium assessment in elderly patients (24). The diagnosis of postoperative delirium required the presence of feature 1 (acute change or fluctuating course), feature 2 (inattention), plus either feature 3 (altered level of consciousness) or feature 4 (disorganized thinking). In addition, patients were evaluated only once within 24 h after surgery. During the 2nd to 7th day after surgery, the patients were assessed twice a day (8:00–10:00 and 18:00–20:00). These evaluations were conducted independently and non-simultaneously by two members of the research team, who were blinded to one another’s assessment findings. Furthermore, to capture delirium episodes occurring outside these scheduled assessment times, medical record reviews were performed, with supplementary confirmation of POD diagnoses based on attending physicians’ documentation in the electronic health record system.

### Statistical analysis and sample size

Based on existing literature (4, 5, 25), sample size was calculated using observational study methodology, assuming an expected POD incidence of 35%. Given the inclusion of 6 independent variables (requiring 10 events per variable [EPV]), the sample size was estimated using the formula: *n* = (Number of Variables × Events per Variable)/Incidence Rate = (6 × 10)/0.35 (26), yielding a minimum required sample size of 172 subjects. To account for a projected 15% attrition rate, 203 participants were consecutively enrolled initially. Due to three patients being immediately admitted to the ICU after surgery, 200 participants were ultimately included in the final analysis.

Statistical analysis was performed using IBM SPSS Statistics software (version 28.0). The normality of the variable’s distribution was analyzed using the Kolmogorov-Smirnov test. Continuous variables with a normal distribution were presented as mean ± standard deviation (SD). Categorical variables were expressed as numbers and percentages. Student’s *t-*test and the Mann-Whitney *U* test were used for continuous variables, and the Pearson chi-square test and Fisher’s exact test were used for categorical variables. All clinically relevant and statistically significant preoperative variables were then entered into a multivariate logistic regression analysis using a forward entry method to identify independent preoperative risk factors for POD. A subgroup analysis included only preoperative chronic pain patients. It was carried out using the same procedure as described above. To further evaluate the robustness of the anesthesia finding in subgroup, we performed a post-hoc power calculation. A *P* < 0.05 was considered statistically significant in all analyses.

## Results

### Characteristics of participants

**FIGURE 1:**
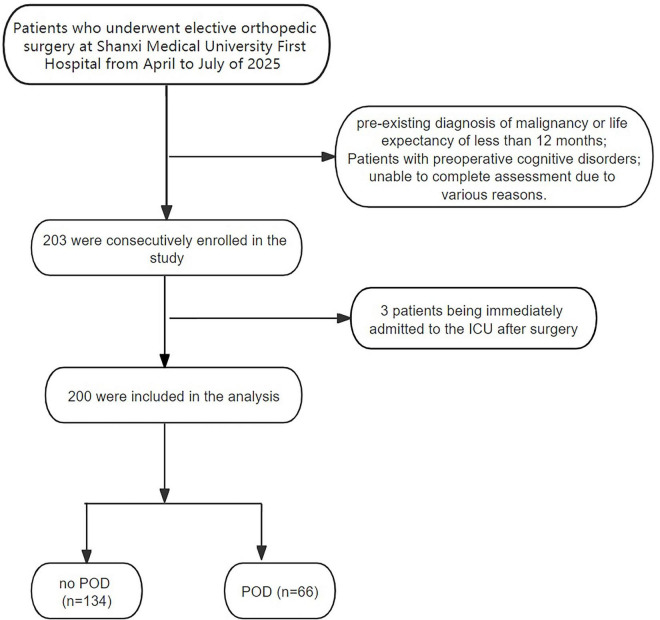
Flow chart of included population.

A total of 203 patients were enrolled. Three were excluded, leaving 200 patients for analysis (Figure 1). The median age of the participants was 69 years (Mean ± SD: 71.12 ± 7.01 years), with a mean BMI of 24.55 ± 3.53 kg/m^2^. and the majority were ASA II (53%) and III (47%). The overall incidence of POD was 33% (*n* = 66), and preoperative chronic pain was present in 58.5% (*n* = 117) of patients. The mean VAS score was 4.01 ± 3.55. The mean operative time, anesthesia time, and intraoperative blood loss were 112.69 ± 38.61 min, 147.29 ± 42.27 min, and 129.25 ± 67.64 ml, respectively. Comparative analyses (Table 1) showed that, compared with non-POD patients, POD patients were significantly older (*P* = 0.012), had poorer physical status (*P* = 0.016), experienced greater intraoperative blood loss (*P* = 0.015), had a higher prevalence of preoperative chronic pain (*P* = 0.024), and reported more severe preoperative pain intensity (*P* = 0.002). In contrast, no significant between-group differences were observed (all *P* > 0.05) for variables including BMI, smoking history, alcohol consumption, educational level, surgical duration, anesthesia duration, PACU length of stay, and use of postoperative patient-controlled analgesia (PCA) pumps.

**TABLE 1:** Baseline characteristics of the study population.

Variables	Total (*n* = 200)	No POD (*n* = 134)	POD (*n* = 66)	Statistic	*P*
Age, Mean ± SD	71.12 ± 7.01	70.16 ± 6.24	73.06 ± 8.06	*t* = -2.57	0.012^#^
BMI, Mean ± SD	24.55 ± 3.53	24.62 ± 3.50	24.41 ± 3.63	*t* = 0.40	0.690^#^
Number of comorbidities, Mean ± SD	0.86 ± 1.03	0.81 ± 0.89	0.98 ± 1.27	*t* = -1.16	0.249^#^
VAS, Mean ± SD	4.01 ± 3.55	3.47 ± 3.42	5.12 ± 3.57	*t* = -3.17	0.002^#^
Duration of surgery (min), Mean ± SD	112.69 ± 38.61	113.70 ± 38.66	110.65 ± 38.71	*t* = 0.52	0.601^#^
Duration of anesthesia (min), Mean ± SD	147.29 ± 42.27	147.67 ± 43.58	146.52 ± 39.78	*t* = 0.18	0.856^#^
Blood loss during surgery (mL), Mean ± SD	129.25 ± 67.64	120.15 ± 58.43	147.73 ± 80.67	*t* = -2.48	0.015^#^
PACU (min), Mean ± SD	26.54 ± 6.14	26.56 ± 6.13	26.50 ± 6.21	*t* = 0.06	0.949^#^
Alcohol, n (%)				χ^2^ = 0.81	0.368*
No	156 (78.00)	107 (79.85)	49 (74.24)		
Yes	44 (22.00)	27 (20.15)	17 (25.76)		
Smoking, n (%)				χ^2^ = 0.06	0.802*
No	167 (83.92)	111 (83.46)	56 (84.85)		
Yes	32 (16.08)	22 (16.54)	10 (15.15)		
Education, n (%)				–	0.633 ^+^
Junior middle school	94 (47.00)	60 (44.78)	34 (51.52)		
Senior high school	105 (52.50)	73 (54.48)	32 (48.48)		
Undergraduate	1 (0.50)	1 (0.75)	0 (0.00)		
Pain, n (%)				χ^2^ = 5.09	0.024*
Not present	83 (41.50)	63 (47.01)	20 (30.30)		
Present	117 (58.50)	71 (52.99)	46 (69.70)		
ASA, n (%)				χ^2^ = 5.78	0.016*
II	106 (53.00)	79 (58.96)	27 (40.91)		
III	94 (47.00)	55 (41.04)	39 (59.09)		
Anesthesia, n (%)				χ^2^ = 2.07	0.150*
Spinal anesthesia	51 (25.50)	30 (22.39)	21 (31.82)		
Total intravenous anesthesia	149 (74.50)	104 (77.61)	45 (68.18)		
Pcia, n (%)				χ^2^ = 1.51	0.220*
Not use	63 (31.50)	46 (34.33)	17 (25.76)		
Use	137 (68.50)	88 (65.67)	49 (74.24)		

BMI, body mass index; PACU, Post Anesthesia Care Unit; ASA, American Society of Anesthesiologists Physical Status Classification; Pcia, Patient Controlled Intravenous Analgesia. These analyses were performed using *Chi-square test, ^#^Mann-Whitney U-test and ^+^Fisher’s exact tests.

### Univariate and multivariate analysis of POD predictors

**FIGURE 2:**
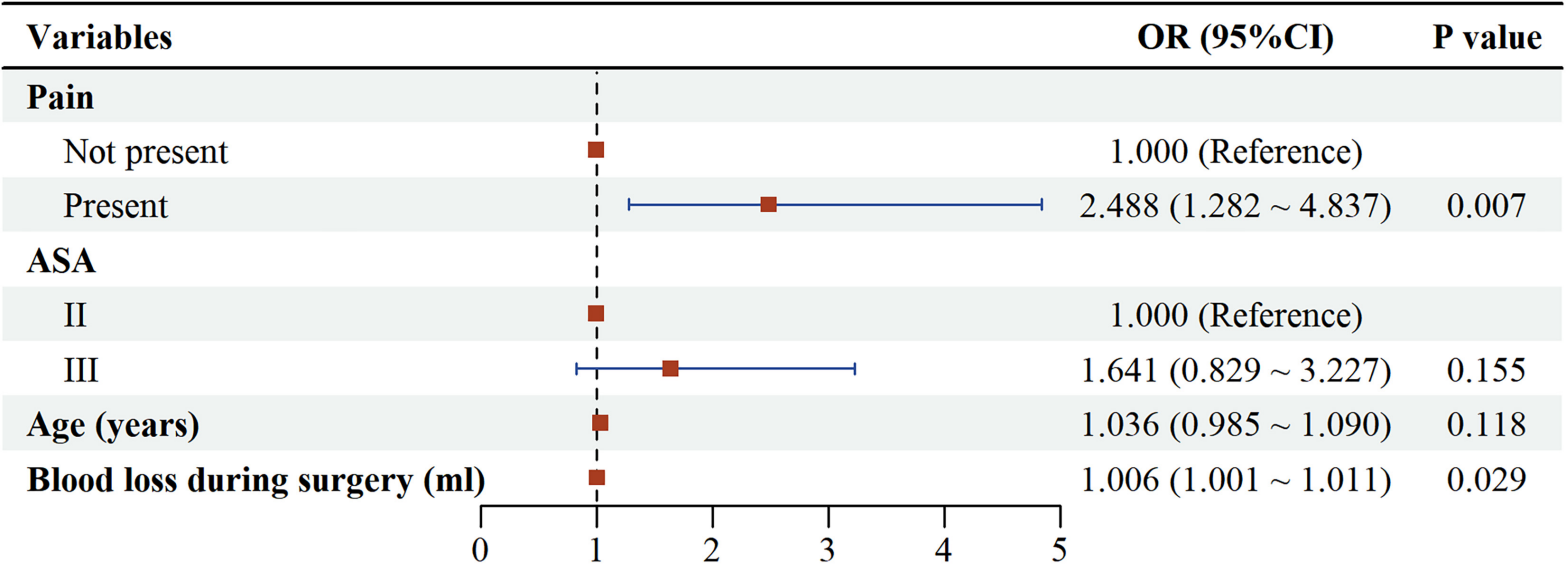
Forest plot. For categorical variables, the reference groups are: Pain (Not present), ASA (II).

Univariate logistic regression results are shown in Table 2. Several factors emerged as statistically significant univariate risk factors, these included preoperative chronic pain (OR = 2.039, 95% CI: 1.090–3.811, *P* = 0.025), higher VAS scores (OR = 1.153, 95% CI: 1.052–1.250, *P* = 0.002), elevated ASA PS (OR = 2.072, 95% CI: 1.143–3.778, *P* = 0.017), advanced age (OR = 1.064, 95% CI: 1.018–1.104, *P* = 0.007), and increased intraoperative blood loss (OR = 1.010, 95% CI: 1.008–1.014, *P* = 0.009). In contrast, no significant associations were observed for educational level, anesthesia type, comorbidity count, surgical duration, anesthesia duration, or PACU length of stay. Based on these findings, preoperative chronic pain, ASA PS, age, and intraoperative blood loss were included in multivariable regression models (Figure 2 and Table 2). The results ultimately revealed that preoperative chronic pain was an independent risk factor significantly associated with POD (OR = 2.488, 95% CI: 1.282–4.837, *P* = 0.007), while intraoperative blood loss emerged as a potential synergistic contributor to POD risk. Besides, the independent effects of age and ASA PS attenuated after covariate adjustment and lost statistical significance.

**TABLE 2:** Univariate and multivariate logistic regression results.

Variables	Univariate		Multivariate	
	OR (95%CI)	P	OR (95%CI)	P
Education				
Junior middle school	1.000 (Reference)	0.395		
Senior high school	0.770 (0.427 ∼ 1.401)			
Undergraduate	0.000 (0.000 ∼ Inf)	0.987		
Pain				
Not present	1.000 (Reference)	0.025	1.00 (Reference)	0.007
Present	2.039 (1.090 ∼ 3.811)		2.488 (1.282 ∼ 4.837)	
ASA				
II	1.000 (Reference)	0.017	1.00 (Reference)	0.155
III	2.072 (1.143 ∼ 3.778)		1.641 (0.829 ∼ 3.227)	
Anesthesia				
Spinal anesthesia	1.000 (Reference)	0.152		
Total intravenous anesthesia	0.623 (0.322 ∼ 1.185)			
Pcia				
No	1.000 (Reference)	0.221	1.036 (0.985∼1.090)	0.118
Yes	1.512 (0.779 ∼ 2.912)			
Age (years)	1.064 (1.018 ∼ 1.104)	0.007		
Number of comorbidities	1.180 (0.887 ∼ 1.558)	0.251		
VAS	1.153 (1.052 ∼ 1.250)	0.002	1.771 (1.260 ∼ 2.487)	< 0.001
Duration of surgery (min)	1.001 (0.986 ∼ 1.014)	0.599		
Duration of anesthesia (min)	1.002 (0.992 ∼ 1.009)	0.855		
Blood loss during surgery (mL)	1.010 (1.008 ∼ 1.014)	0.009	1.006 (1.001 ∼ 1.011)	0.029
PACU (min)	1.002 (0.948 ∼ 1.053)	0.948		

PACU, Post Anesthesia Care Unit; ASA, American Society of Anesthesiologists Physical Status Classification; Pcia, Patient Controlled Intravenous Analgesia.

### Subgroup analysis

The pain-specific subgroup analysis included 117 patients (58.5%) with preoperative chronic pain. Among these patients, comparisons of baseline characteristics (Table 3) showed that the cohort with delirium was older (*P* = 0.020), had higher VAS scores (*P* = 0.002), had a higher proportion of ASA PS 3 (*P* = 0.05), and had a higher prevalence of non-general anesthesia (*P* = 0.004).

**TABLE 3:** Baseline characteristics of subgroup.

Variables	Total (*n* = 117)	NO POD (*n* = 71)	POD (*n* = 46)	Statistic	*P*
Age, Mean ± SD	70.64 ± 6.57	69.51 ± 5.75	72.39 ± 7.40	*t* = –2.36	0.020^#^
BMI, Mean ± SD	25.06 ± 3.62	25.33 ± 3.34	24.64 ± 4.01	*t* = 1.02	0.312^#^
Number of comorbidities, Mean ± SD	0.85 ± 1.02	0.75 ± 0.84	1.00 ± 1.25	*t* = –1.31	0.191^#^
VAS, Mean ± SD	6.86 ± 1.36	6.55 ± 1.32	7.35 ± 1.29	*t* = –3.23	0.002^#^
Duration of surgery (min), Mean ± SD	111.02 ± 37.67	111.90 ± 35.90	109.65 ± 40.61	*t* = 0.31	0.754^#^
Duration of anesthesia (min), Mean ± SD	142.93 ± 37.61	142.62 ± 36.89	143.41 ± 39.11	*t* = –0.11	0.912^#^
Blood loss during surgery (mL), Mean ± SD	124.27 ± 70.02	113.94 ± 59.22	140.22 ± 82.18	*t* = –1.88	0.065^#^
PACU (min), Mean ± SD	26.77 ± 6.24	26.79 ± 6.17	26.74 ± 6.40	*t* = 0.04	0.967^#^
Alcohol, n (%)				χ^2^ = 2.49	0.115*
No	97 (82.91)	62 (87.32)	35 (76.09)		
Yes	20 (17.09)	9 (12.68)	11 (23.91)		
Smoking, n (%)				χ^2^ = 0.62	0.431*
No	97 (83.62)	57 (81.43)	40 (86.96)		
Yes	19 (16.38)	13 (18.57)	6 (13.04)		
Education, n (%)				–	0.664 ^+^
Junior middle school	50 (42.74)	28 (39.44)	22 (47.83)		
Senior high school	66 (56.41)	42 (59.15)	24 (52.17)		
Undergraduate	1 (0.85)	1 (1.41)	0 (0.00)		
ASA, n (%)				χ^2^ = 3.85	0.050*
2	64 (54.70)	44 (61.97)	20 (43.48)		
3	53 (45.30)	27 (38.03)	26 (56.52)		
Anesthesia, n (%)				χ^2^ = 8.12	0.004*
Spinal anesthesia	25 (21.37)	9 (12.68)	16 (34.78)		
Total intravenous anesthesia	92 (78.63)	62 (87.32)	30 (65.22)		
Pcia, n (%)				χ^2^ = 1.07	0.300*
No	37 (31.62)	25 (35.21)	12 (26.09)		
Yes	80 (68.38)	46 (64.79)	34 (73.91)		

BMI, body mass index; PACU, Post Anesthesia Care Unit; ASA, American Society of Anesthesiologists Physical Status Classification; Pcia, Patient Controlled Intravenous Analgesia. These analyses were performed using *Chi-square test, ^#^Mann-Whitney *U*-test and ^+^Fisher’s exact tests.

**FIGURE 3:**
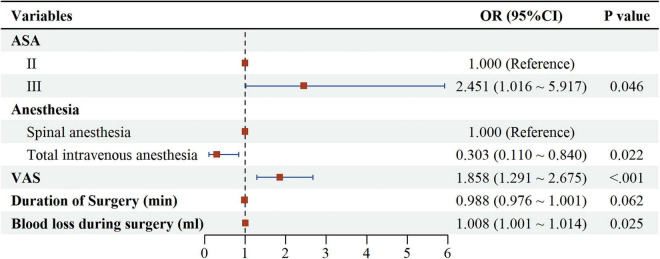
Forest plot of subgroup. For categorical variables, the reference groups are: ASA (II), Anesthesia (Spinal Anesthesia).

Ultimately, multivariable logistic regression analyses (Figure 3 and Table 4) identified three independent risk factors for postoperative delirium in this chronic pain subpopulation: higher ASA PS (OR = 2.451, 95% CI: 1.016–5.917, *P* = 0.046), elevated VAS scores (OR = 1.858, 95% CI: 1.291–2.675, *P* < 0.001), and increased intraoperative blood loss (OR = 1.008, 95% CI: 1.001–1.014, *P* = 0.025). In contrast, general anesthesia showing a significant association with lower odds of POD compared to spinal anesthesia (OR = 0.303, 95% CI: 0.110–0.840, *P* = 0.022). *Post hoc* power calculation for the anesthesia comparison, based on observed POD rates [spinal: 64% (16/25) vs. general: 32.6% (30/92)] and α = 0.05, yielded approximately 81% power.

**TABLE 4:** Univariate and multivariate logistic regression results in subgroup.

	Univariate		Multivariate	
Variables	OR (95%CI)	*P*	OR (95%CI)	*P*
Education				
Junior middle school	1.000 (Reference)	0.406		
Senior high school	0.732 (0.344 ∼ 1.538)			
Undergraduate	0.000 (0.000 ∼ Inf)	0.992		
ASA				
II	1.000 (Reference)	0.051	1.00 (Reference)	0.046
III	2.122 (1.002 ∼ 4.514)		2.451 (1.016 ∼ 5.917)	
Anesthesia				
Spinal anesthesia	1.000 (Reference)	0.006	1.00 (Reference)	0.022
Total intravenous anesthesia	0.267 (0.114 ∼ 0.687)		0.303 (0.110 ∼ 0.840)	
Pcia				
No	1.000 (Reference)	0.301		
Yes	1.544 (0.676 ∼ 3.492)			
Age (years)	1.068 (1.013 ∼ 1.132)	0.024		
Number of comorbidities	1.281 (0.883 ∼ 1.842)	0.197		
VAS	1.612 (1.176 ∼ 2.188)	0.003	1.858 (1.291 ∼ 2.675)	< 0.001
Duration of surgery (min)	1.002 (0.993 ∼ 1.014)	0.752	0.988 (0.976 ∼ 1.001)	0.062
Duration of anesthesia (min)	1.002 (0.994 ∼ 1.016)	0.911		
Blood loss during surgery (mL)	1.008 (1.003 ∼ 1.017)	0.055	1.008 (1.001∼ 1.014)	0.025
PACU (min)	1.003 (0.938 ∼ 1.057)	0.966		

BMI, body mass index; PACU, Post Anesthesia Care Unit; ASA, American Society of Anesthesiologists Physical Status Classification; Pcia, Patient Controlled Intravenous Analgesia.

## Discussion

Our findings demonstrated that preoperative chronic pain is significantly and independently associated with the development of POD. Specifically, increasing pain intensity is associated with a progressively higher risk of delirium in a dose-dependent manner. Additionally, we identified that advanced age, higher ASA PS, increased intraoperative blood loss, and anesthesia modality further contribute to susceptibility to POD. Notably, subgroup analysis of patients with chronic pain suggested that general anesthesia may exert a protective effect against the onset of delirium, though these findings must be interpreted with caution due to the potential confounding influences.

Many studies have demonstrated that chronic pain impaired memory (27–29). Guusje and colleagues found that high levels pain led to cognitive decline and that elderly adults with chronic pain had a higher risk of developing cognitive impairment (30). These studies primarily focused on high-risk populations (e.g., older adults, those with preexisting cognitive vulnerabilities, or undergoing complex surgeries) who are inherently susceptible to chronic pain-related cognitive impairment and perioperative neurological complications. While prior studies investigating risk factors for POD in non-cardiac surgical populations have identified inadequately managed postoperative pain as a modifiable risk factor (18, 31), preoperative chronic pain has not been consistently validated as a predictor of POD (22, 23). This discrepancy can be partially attributed to the inclusion of low-risk populations (e.g., younger adults with minimal comorbidities, minor surgeries) in negative studies, where chronic pain burden may not reach the threshold for POD onset, and also the impact of preoperative chronic pain on delirium may be diluted. However, our study mainly included elderly patients with comorbidities, lowers the difficulty to observe the association between the two. The present study confirms the key clinical significance of preoperative chronic pain as an intervenable risk factor, demonstrating that both chronic pain status and VAS scores independently predict POD, highlighting the critical role of population risk stratification, the association is more likely detected in high-risk cohorts (32), where pain-related cognitive disruption synergizes with perioperative stressors (e.g., anesthesia, inflammation) to amplify POD risk. This aligns with Pisani’ findings (25) of pain intensity is particularly pronounced in high-risk groups.

The observed association may be explained by several potential pathways proposed in the literature. One hypothesized mechanism involves nociception-triggered neuroinflammatory cascades, and the other proposed pathway relates to preoperative chronic pain. For the former, animal studies have provided supportive evidence, demonstrating that noxious stimuli can upregulate the expression of interleukin-1β (IL-1β) in the hippocampus, impair the integrity of the blood-brain barrier, and activate microglia—these pathological changes collectively induce delirium-like phenotypes, which are closely associated with the development of cognitive impairment (33). For the latter, preoperative chronic pain may facilitate the impairment of cognitive function in patients after anesthesia administration through the periaqueductal gray-dorsal raphe (PAG-DR) neural circuit. Given these potential pathways, preventative analgesia has been proposed as a considerable and targeted measure to reduce the incidence of POD (34).

Many other factors might contribute to the easier development of POD in patients with preoperative chronic pain. The ASA PS as a key preoperative indicator of underlying disease severity, primarily reflecting impaired systemic health and multiple comorbidities. Our univariate analysis showed that patients with ASA III had higher risk of delirium compared to those with ASA II, with this risk being particularly pronounced in the pain subgroup, which consistent with prior epidemiological evidence (35). This phenomenon is likely due to synergistic interactions between comorbidities, which increase baseline vulnerability in elderly patients and ultimately result in POD when combined with surgical stress. Advanced age is a well-established determinant of various postoperative complications. Our analysis showed a finding consistent with prior evidence identifying patients aged ≥ 79 years as particularly vulnerable to POD (36). This heightened susceptibility likely arises from age-related declines in physiological reserve across organ systems. We also identify intraoperative blood loss as a significant risk factor for POD in elderly orthopedic patients, with consistent predictive value across analyses. Intraoperative blood loss showed a stable, consistent association across all analytical models, a finding consistent with prior research (37). The proposed pathophysiological mechanisms involve complex multi-pathway interactions. These mechanisms primarily include impaired cerebral energy metabolism, exacerbation of frailty syndrome, and activation of neuroinflammatory cascades. Specifically, intraoperative hemorrhage reduces circulating blood volume, resulting in inadequate cerebral oxygen delivery, which in turn induces mitochondrial dysfunction and diminished adenosine triphosphate (ATP) synthesis (38).

Subgroup analysis revealed that among patients with pre-existing chronic pain, those receiving spinal anesthesia had significantly higher POD risk than those undergoing general anesthesia, even after adjusting for age, ASA PS and blood loss. However, this finding must be interpreted with extreme caution due to potential confounding by indication. As shown in Supplementary Table 1, patients selected for spinal anesthesia were significantly older, had more comorbidities, and exhibited higher baseline POD risk—systematic differences that likely influenced both anesthesia choice and outcomes. Although we adjusted for measured confounders, residual confounding from unmeasured factors (e.g., frailty, surgical complexity) cannot be excluded. The wide confidence interval (OR = 0.303, 95% CI: 0.110–0.840) reflects imprecision stemming from the small sample size. While a *post hoc* power calculation suggested adequate power (81%), this estimate relies on the observed effect size, which is itself unstable. Notably, this finding differs from previous studies reporting no association between anesthetic technique and POD (39–41), further underscoring its preliminary nature. Therefore, this result should not inform clinical practice but rather serve as a hypothesis-generating observation warranting validation in large-scale, multicenter randomized controlled trials designed to examine the interaction between preoperative pain and anesthetic technique on POD.

This study revealed a high prevalence of pre-existing chronic pain in the cohort. Of the 200 enrolled participants, 117 (58.5%) reported chronic pain prior to surgery—a proportion significantly higher than the anticipated prevalence (42). This high rate may stem from two key factors. First, our study specifically included elderly patients ( ≥ 65 years) undergoing orthopedic surgery. As noted in the introduction, this population has a heightened predisposition to comorbid chronic pain conditions. Second, the operational definition of chronic pain used in this study likely had broader inclusion criteria compared to other established definitions. Participants were classified as having pre-existing chronic pain solely based on self-reported pain symptoms lasting ≥ 3 months. Relying exclusively on symptom duration as the diagnostic criterion may have led to misclassification of some individuals as having chronic pain, even if they did not meet other diagnostic requirements. Such non-differential misclassification would likely bias our results toward the null, meaning the true association between clinically significant chronic pain and POD could be even stronger than the one we observed.

Furthermore, our assessment of preoperative chronic pain, while capturing duration and intensity via VAS, did not incorporate a detailed characterization of pain quality (e.g., neuropathic vs. nociceptive), etiology (e.g., osteoarthritis vs. inflammatory arthritis), or pattern. These factors could potentially influence the physiological stress response and subsequent delirium risk differently. Future studies with more granular pain phenotyping are warranted to explore these potential differential effects.

This study is the first to systematically explore the association between pre-existing chronic pain and POD, specifically in elderly orthopedic surgical patients. Prior research focused mainly on non-cardiac surgical cohorts and rarely distinguished acute from chronic pain, thus our findings fill a key knowledge gap in this high-risk population. Notably, we also first identified a potential protective effect of general anesthesia against POD in patients with preoperative chronic pain. This novel observation indicates a significant interaction between pain status and anesthetic modality, providing a basis for new intervention strategies targeting the “pain-anesthesia interaction” pathway, which warrants further exploration as a promising direction for future research.

The primary limitation of this study lies in its relatively small sample size, which reduced the reliability of subgroup analyses. For example, the observed protective effect of anesthetic modality was accompanied by a wide confidence interval spanning unity, indicating limited precision and stability of this finding. Importantly, we were unable to adjust for several well-established confounders of POD, including preoperative electrolyte imbalances, nutritional status, and postoperative pain scores or ambulatory status. The omission of these potentially important variables could have introduced residual confounding, which might have influenced the observed association between chronic pain and POD. Future research should incorporate a more comprehensive set of covariates to better isolate the independent effect of preoperative chronic pain.

## Conclusion

This prospective cohort study provides evidence that preoperative chronic pain is a modifiable, independent risk factor for POD in geriatric orthopedic surgical populations. Notably, its modifiability identifies tangible targets for clinical interventions. Concurrently, intraoperative blood loss emerged as a variable with robust, generalizable predictive utility, serving as a reliable perioperative warning indicator across diverse clinical scenarios in this high-risk population. Despite these clinically meaningful observations, the current findings require rigorous external validation via larger-scale, multi-center studies to confirm their reproducibility and generalizability.

## Data Availability

The original contributions presented in this study are included in this article/Supplementary material, further inquiries can be directed to the corresponding authors.
