# Supplementary material for: The association between preoperative chronic pain and postoperative delirium in elderly patients undergoing elective orthopedic surgery
Source: Front Med (Lausanne). 2026 Mar 23;13:1795717. doi: 10.3389/fmed.2026.1795717 (PMC13050949; doi:10.3389/fmed.2026.1795717)
Supplement: Supplementary file 1 [file Table_1.docx]

Supplementary Table 1

| Variables | Total (n = 117) | SA (n = 25) | TIVA (n = 92) | Statistic | *P* |
| --- | --- | --- | --- | --- | --- |
|  |  |  |  |  |  |
| Age, Mean ± SD | 70.64 ± 6.57 | 73.76 ± 8.38 | 69.79 ± 5.76 | t=2.23 | 0.033 |
| BMI, Mean ± SD | 25.06 ± 3.62 | 23.69 ± 4.64 | 25.43 ± 3.22 | t=-1.76 | 0.088 |
| Number Of Comorbidities, Mean ± SD | 0.85 ± 1.02 | 1.44 ± 1.42 | 0.68 ± 0.82 | t=3.42 | <.001 |
| VAS, Mean ± SD | 6.86 ± 1.36 | 7.16 ± 1.37 | 6.78 ± 1.35 | t=1.24 | 0.219 |
| Duration Of Surgery (min), Mean ± SD | 111.02 ± 37.67 | 116.52 ± 34.31 | 109.52 ± 38.57 | t=0.82 | 0.412 |
| Duration Of Anesthesia (min), Mean ± SD | 142.93 ± 37.61 | 144.16 ± 35.13 | 142.60 ± 38.44 | t=0.18 | 0.855 |
| Blood loss during surgery(ml), Mean ± SD | 124.27 ± 70.02 | 139.20 ± 83.41 | 120.22 ± 65.85 | t=1.20 | 0.231 |
| PACU (min), Mean ± SD | 26.77 ± 6.24 | 21.04 ± 6.21 | 28.33 ± 5.28 | t=-5.88 | <.001 |
| Alcohol, n (%) |  |  |  | χ²=0.54 | 0.462 |
| No | 97 (82.91) | 19 (76.00) | 78 (84.78) |  |  |
| Yes | 20 (17.09) | 6 (24.00) | 14 (15.22) |  |  |
| Smoking, n (%) |  |  |  | χ²=0.06 | 0.805 |
| No | 97 (83.62) | 20 (80.00) | 77 (84.62) |  |  |
| Yes | 19 (16.38) | 5 (20.00) | 14 (15.38) |  |  |
| Education, n (%) |  |  |  | - | 0.350 |
| Junior middle school | 50 (42.74) | 14 (56.00) | 36 (39.13) |  |  |
| Senior high school | 66 (56.41) | 11 (44.00) | 55 (59.78) |  |  |
| Undergraduate | 1 (0.85) | 0 (0.00) | 1 (1.09) |  |  |
| ASA, n (%) |  |  |  | χ²=0.58 | 0.448 |
| II | 64 (54.70) | 12 (48.00) | 52 (56.52) |  |  |
| III | 53 (45.30) | 13 (52.00) | 40 (43.48) |  |  |
| Pcia, n (%) |  |  |  | χ²=5.66 | 0.017 |
| Not Use | 37 (31.62) | 3 (12.00) | 34 (36.96) |  |  |
| Use | 80 (68.38) | 22 (88.00) | 58 (63.04) |  |  |
| Delirium, n (%) |  |  |  | χ²=8.12 | 0.004 |
| No | 71 (60.68) | 9 (36.00) | 62 (67.39) |  |  |
| Yes | 46 (39.32) | 16 (64.00) | 30 (32.61) |  |  |

t: t-test, χ²: Chi-square test, -: Fisher exact, D: standard deviation;
BMI, body mass index; PACU, Post Anesthesia Care Unit; ASA, American Society of Anesthesiologists Physical Status Classification; Pcia, Patient Controlled Intravenous Analgesia.
